# Effects of *Lactiplantibacillus plantarum* and *Lacticaseibacillus paracasei* supplementation on the faecal metabolome in children with coeliac disease autoimmunity: a randomised, double-blinded placebo-controlled clinical trial

**DOI:** 10.3389/fnut.2023.1183963

**Published:** 2023-07-06

**Authors:** Eliska Jenickova, Carin Andrén Aronsson, Anna Mascellani Bergo, Ondrej Cinek, Jaroslav Havlik, Daniel Agardh

**Affiliations:** ^1^Department of Food Science, Czech University of Life Sciences Prague, Prague, Czechia; ^2^Department of Clinical Sciences, Lund University, Malmö, Sweden; ^3^Department of Pediatrics and Department of Medical Microbiology, Charles University in Prague and University Hospital Motol, Prague, Czechia

**Keywords:** coeliac disease, gut metabolome, *Lactiplantibacillus plantarum*, *Lacticaseibacillus paracasei*, NMR, probiotics

## Abstract

**Introduction:**

Coeliac disease is a lifelong immune-mediated enteropathy manifested as gluten intolerance in individuals carrying specific human leukocyte antigen (HLA) molecules. Other factors than genetics and gluten intake, however, may play a role in triggering the disease. The gut internal environment is thought to be one of these potential contributing factors, and it can be influenced throughout life.

**Methods:**

We examine the impact of Lactiplantibacillus plantarum HEAL9 and Lacticaseibacillus paracasei 8700:2 supplementation on the faecal metabolome in genetically predisposed children having tissue transglutaminase autoantibodies, i.e., coeliac disease autoimmunity. Probiotic strains were selected based on their beneficial properties, including mucosal permeability and immune modulation effects. The intervention group (*n* = 40) and control group (*n* = 38) took the probiotics or placebo daily for 6 months in a double-blinded randomised trial. Faecal samples were collected at baseline and after 3 and 6 months and analysed using the 1H NMR for metabolome. The incorporation of 16S rRNA sequencing as a supportive dataset complemented the analysis of the metabolome data.

**Results:**

During the 6 months of intervention, the stool concentrations of 4-hydroxyphenylacetate increased in the intervention group as compared to controls, whereas concentrations of threonine, valine, leucine, isoleucine, methionine, phenylalanine, aspartate, and fumarate decreased. Additionally, a noteworthy effect on the glycine, serine, and threonine metabolic pathway has been observed.

**Conclusion:**

The findings suggest a modest yet significant impact of the probiotics on the faecal metabolome, primarily influencing proteolytic processes in the gut.

**Clinical trial registration:**

ClinicalTrials.gov, NCT03176095.

## Introduction

Coeliac disease (CD) is a lifelong immune-mediated enteropathy manifested as gluten intolerance in individuals carrying specific human leukocyte antigen (HLA) haplotypes (1). Gluten intolerance occurs as a reaction to dietary gluten mostly from wheat, rye, or barley anytime in life. It arises in a small fraction of gluten-exposed genetically permissible subjects. Its incidence seems to be rising globally (2, 3).

There are indications that the gut microbiome may play a role in CD pathogenesis, progression, and clinical presentation (4–6). Several studies have reported imbalances in the gut microbiome of patients with CD leading to dysbiosis. Studies in the established CD are, however, prone to reverse causation—it is unclear whether these alterations are a symptom of the disease or a contributing factor (6–8). Interventions with probiotics are, therefore, one of the logical strategies for unravelling the role of bacteria in disease pathogenesis.

Probiotics have been suggested as a potential adjunctive therapy for CD (9, 10). Previous trials have mainly tested interventions of genera *Bifidobacterium* and (formerly) *Lactobacillus* (11, 12). Supplementation of *Bifidobacterium breve* has been suggested to decrease pro-inflammatory cytokines and cause positive alterations in the SCFA profiles (13). Even though most of the trials focussed on ongoing CD, changes in the gut environment may occur even before the diagnosis, thus preceding the disease onset and potentially allowing prevention (6, 14). In the present randomised clinical trial, Celiac disease Prevention with Probiotics (CiPP) study (15, 16), children with the persistence of tissue transglutaminase autoantibodies (tTGA), i.e., CD autoimmunity, received *Lactiplantibacillus plantarum* HEAL9 and *Lacticaseibacillus paracasei* 8700:2 probiotics, or placebo, for 6 months. This intervention resulted in alterations in the peripheral lymphocyte immune response, but there was no overall difference in tTGA levels compared with the placebo group (15). Subtle changes upon the intervention were noted in the microbiota, mainly in the abundance of genera *Prevotella, Akkermansia, Bifidobacterium*, and *Streptococcus* (16).

Faecal metabolites are primary products of microbial metabolism, but also reflect factors such as bile and enzyme activity, gut barrier function, transit time, and diet of the host. Nuclear magnetic resonance (NMR) is one of the standard methods used for the analysis of metabolites present in the Faecal sample (17). The metabolite fingerprinting provides a snapshot of the microbiota's functional capacity as an overview of molecules in the intestine (14, 17, 18). The most studied metabolites are short-chain fatty acids (SCFAs), end-products of bacterial saccharolytic activity. Especially in CD, SCFAs are of major interest due to their involvement in immunomodulatory functions such as the production of regulatory T-cells (19). Some studies also reported alterations in SCFA production in CD patients (20–22), even when on a long-term gluten-free diet (23). Other metabolites with altered amounts in CD are glutamine and tryptophan, which also impact the immune system (24, 25).

The aim of the present study was to describe the composition of the faecal metabolome and test its changes associated with the probiotic intervention in the setting of the abovementioned randomised double-blinded clinical trial.

## Materials and methods

### Study participants and sample collection

The CiPP study recruited 78 children aged 2–11 years with ongoing CD autoimmunity, i.e., defined as screening-identified persistent positivity for tTGA in two consecutive samples, enrolled between March 2012 and August 2015 (15). The enrolled children were identified among carriers of HLA-genotypes associated with CD (DR3-DQ2/DR3-DQ2, DR3-DQ2/DR4-DQ8, DR4-DQ8/DR4-DQ8, and/or DR4-DQ8/DR8-DQ4).

Participating children were invited to a randomisation and baseline visit (visit 0) and scheduled for follow-up visits ~3 (visit 1) and 6 (visit 2) months later. Participants were randomised at a 1:1 ratio to either placebo or intervention group. Among the 78 enrolled children (placebo, *n* = 38; intervention, *n* = 40) (characteristics listed in Table 1), 63 (81%) provided faeces samples for all three visits.

**Table 1:** Baseline characteristics of study participants.

		**Placebo**	**Intervention**	**Total**	*p*-value^**^
Gender	Females	24	18	42	0.1904
	Males	14	22	36	0.0593
	Total	38	40	78	0.1079
Age (years)^*^	Females	5.4 ± 0.5	5.9 ± 0.5	5.6 ± 0.3	0.4179
	Males	4.3 ± 4.3	4.7 ± 0.4	4.5 ± 0.3	0.7490
	Total	5.0 ± 0.3	5.2 ± 0.3	5.1 ± 0.2	0.6384
Weight percentile for a given age (WHO)^*^	Females	68 ± 4.6	71 ± 6.6	69 ± 3.8	0.4237
	Males	73 ± 5.2	76 ± 5.5	75 ± 3.9	0.3320
	Total	70 ± 3.4	74.0 ± 4.2	72 ± 2.7	0.1556
Use of dietary supplements or foods fortified with probiotics before the study starts - yes		19	18	37	0.6585

* Data are presented as mean ± s.d.

** Wilcoxon test or χ-square test.

The study product was an equal mixture of *Lactiplantibacillus plantarum* HEAL9, formerly classified as *Lactobacillus plantarum*, and *Lacticaseibacillus paracasei* 8700:2, formerly classified as *Lactobacillus paracasei*, in a total bacterial dose of 1 × 10^10^ CFU/sachet in maltodextrin (1.0 g). The placebo product consisted of maltodextrin only. The combination of the two *Lactobacillus* strains was chosen due to their different physiological effects, i.e., *Lactiplantibacillus plantarum* HEAL9 targets the permeability of the mucosa, and *Lactocaseibacillus paracasei* 8700:2 targets the immune system (26, 27). All enrolled children followed a regular gluten-containing diet during the study.

Stool sample collection was carried out at home by the study participant's caregiver. Samples were stored at −20°C until they were transported to the lab, where they were kept at −80°C until the analysis. The faecal microbiome was previously analysed using 16S rDNA sequencing (16). The ensuing sequencing data were reprocessed for this study, using the DADA2 pipeline (28) with the non-redundant Silva database version 138 (29).

### Faecal metabolome

The faecal aliquots were prepared accordingly to Jaimes et al. (30). All chemicals and reagents used were of analytical grade and were purchased from Sigma-Aldrich (Merck, Darmstadt, DE). The ^1^H NMR spectra were recorded on a Bruker Avance III HD spectrometer equipped with a broadband fluorine observation SmartProbe^TM^ with z-axis gradients (Bruker BioSpin GmbH, Rheinstetten, Germany) operating at the proton frequency of 500.18 MHz. All samples were acquired using a 1D NOESY pulse sequence with presaturation and calibrated to the internal standard (3-(trimethylsilyl) propionic-2,2,3,3-*d*_4_ acid sodium salt) at 0.0 ppm, manually phased in TopSpin 3.6.4 (Bruker Biospin GmbH, Rheinstetten, Germany). The spectra were pre-processed with an in-house script under MATLAB^®^ R2020a (MathWorks, Natick, MA, USA) consisting of multipoint baseline correction in user-defined segments, ensuring the same pre-processing for all the spectra. Spectra between δ 0.5 and 9.0 ppm (excluding the residual water region, δ 5.1–4.6 ppm) were reduced into defined buckets; each bin representing a spin system or a part of a spin system that was ideally pure, distinct, and quantitative—in most cases, one bin for each metabolite. Ranges for the bins were chosen after annotation of a subset of spectra in the software Chenomx ver. 8.6, using the built-in spectral library, our in-house database, and published annotated stool spectra (30–33). A detailed description of the workflow is shown in Supplementary Figure 1. For statistical processing, buckets were normalised using probabilistic quotient normalisation (34).

### Statistical and multi-component analysis

The metabolome was evaluated using the principal components analysis (PCA) algorithm implemented in PLS-Toolbox 8.9 (Eigenvector Research, Wenatchee, WA, USA) under MATLAB^®^ R2020a environment including buckets of annotated and unknown peaks. PCA was run with concentrations of all annotated and unknown peaks identified in the spectrum, while all other tests were run using only data of annotated features. Linear mixed-effects models were built to characterise the changes in individual metabolite abundance during the three visits; only the annotated buckets were included. The models included individuals as random effect varying intercept only, and the fixed effects were clinic visits and intervention arm. The interaction term between clinic visits and interventions was considered of interest with the aim of showing the impact of dietary intervention in time. The analysis was carried out using the package 'lmerTest' v. 3.1–3 (35) in R v. 4.2.1 (36). Additionally, the Wilcoxon sum rank test and the Wilcoxon signed-rank sum test were applied for pairwise comparison between interventions at each visit as well as for comparison within visit for each intervention, for the subjects who completed all three visits. Correlations between faecal metabolome and faecal microbiome were tested using Spearman's rank correlation test considering ρ > |0.5| using family taxonomic levels that were detected at least in 40% of the samples; the correlations were visualised as a heatmap using Euclidean distance for clustering. Associations between metabolome and 16S rRNA microbiome profiles were analysed on paired metabolome-microbiome datasets of all samples regardless of the group and visit. Only samples from children that provided faecal samples at all clinic visits (visit 0, visit 1, and visit 2) were used for the Wilcoxon tests. After the exclusion, 189 samples (*n* = 63) were compared (intervention group, n=32; placebo group, *n* = 31). For all other analyses, all faecal samples were included (intervention group at visit 0, *n* = 38; intervention group at visit 1, *n* = 37; intervention group at visit 2, *n* = 35; placebo group at visit 0, *n* = 36; placebo group at visit 1, *n* = 34; placebo at visit 2, *n* = 36). Furthermore, to uncover physiological patterns, a pathway analysis between the groups at visit 2 was conducted using MetaboAnalyst 5.0 and the KEGG metabolic library using the genus *E. coli* as a model organism and a proxy for the whole gut microbiota (37, 38). The analysis was performed considering the metabolite overrepresentation within a pathway using the global test, and the influence of changed metabolites on the pathway's function through relative betweenness centrality.

## Results

### Changes in metabolome related to probiotic administration

**Figure 1:**
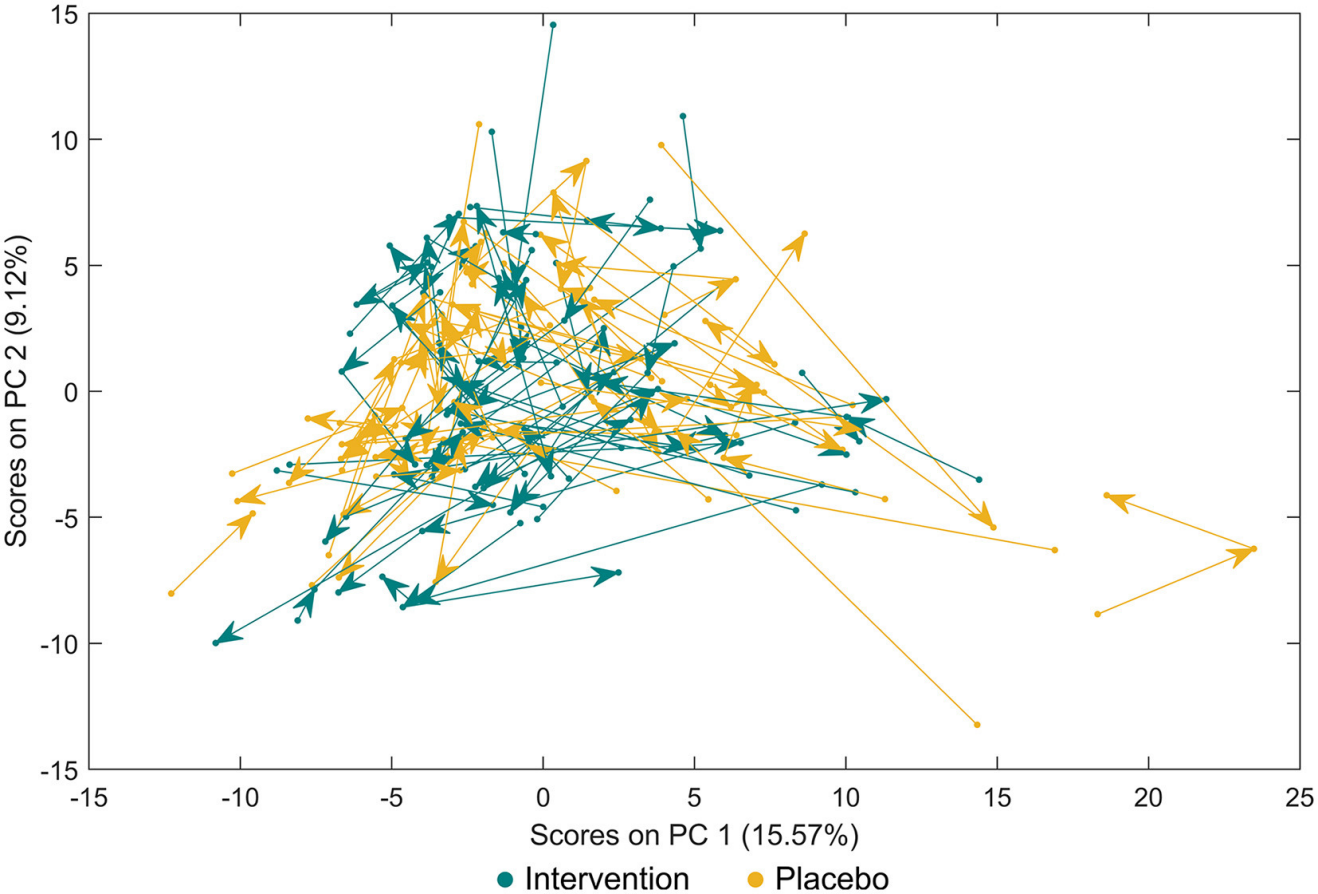
Principal component analysis for faecal metabolites between study participants either receiving a mixture of *Lactiplantibacillus plantarum* HEAL9 and *Lacticaseibacillus paracasei* 8700:2 (intervention) or placebo. Paired data are connected with arrows from the baseline towards scheduled follow-up visits after 3 months and 6 months.

**Figure 2:**
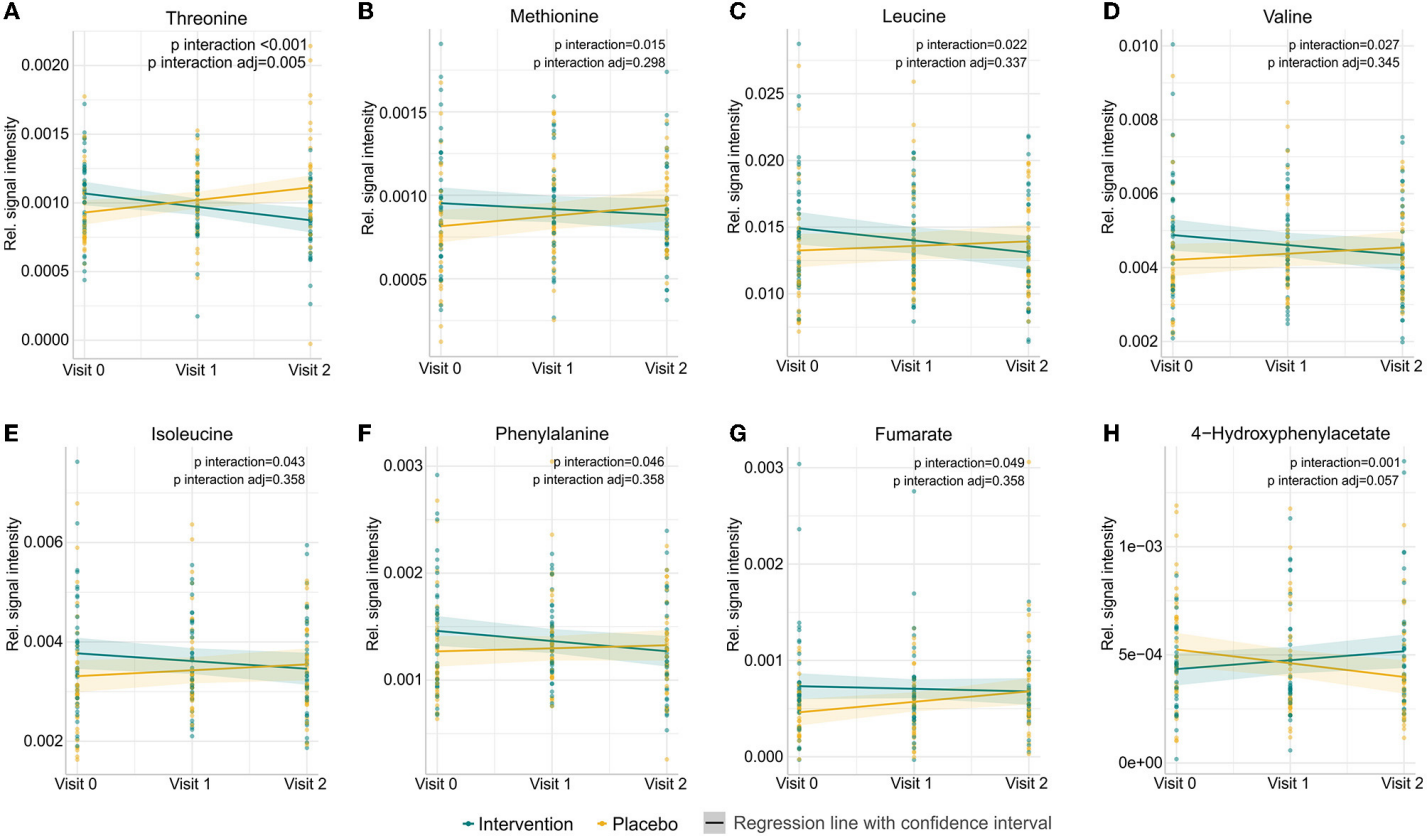
Prediction of changes in abundances of the eight metabolites in participants either receiving a mixture of *Lactiplantibacillus plantarum* HEAL9 and *Lacticaseibacillus paracasei* 8700:2 (intervention) or placebo from the beginning of the study (visit 0), to 3 months (visit 1) and 6 months (visit 2). Significant metabolites identified by linear mixed-effects models showing the impact of dietary intervention in time: **(A)** threonine, **(B)** methionine, **(C)** leucine, **(D)** valine, **(E)** isoleucine, **(F)** phenylalanine, **(G)** fumarate, and **(H)** 4-hydroxyphenylacetate. Raw *p*-values and *p*-values adjusted after the Benjamini–Hochberg correction are shown.

Forty-six metabolites were identified using a semi-targeted approach covering primarily molecules of short-chain fatty acids, branched-chain fatty acids, amino acids, and sugars. The most abundant metabolites were propylene glycol, acetate, butyrate, leucine, and alanine. Additionally, 145 unknown spectral features were uncovered (Supplementary Table 1). An exploratory data approach based on PCA was used as a first step to assess the progress of individuals through the study. Neither common tendency nor clear distinction between the subjects based on the placebo or intervention group was observed at the baseline, visit 1, and visit 2 (Figure 1; Supplementary Figures 2A–C). Therefore, a PCA model showing subjects at visit 1 and visit 2 after having subtracted the registered concentrations at visit 0 on a subject-by-subject basis was considered to highlight the trends possibly hidden by the differences naturally occurring among subjects. No common tendency was observed (Supplementary Figure 2D). On the other hand, the multilevel modelling showed a significant association (*p* < 0.05) of the interaction between time (visits) and the intervention and placebo with eight faecal metabolites (Figure 2). These included a decrease mean slope in the intervention group of threonine (*p* = 6.9 × 10^−5^, Figure 2A), methionine (*p* = 0.015, Figure 2B), leucine (*p* = 0.022, Figure 2C), valine (*p* = 0.027, Figure 2D), isoleucine (*p* = 0.043, Figure 2E), phenylalanine (*p* = 0.046, Figure 2F), and marginally fumarate (*p* = 0.049, Figure 2G), and a positive slope for the placebo group (Figure 2). Oppositely, 4-hydroxyphenylacetate (*p* = 0.001, Figure 2H) increased within the intervention group.

**Figure 3:**
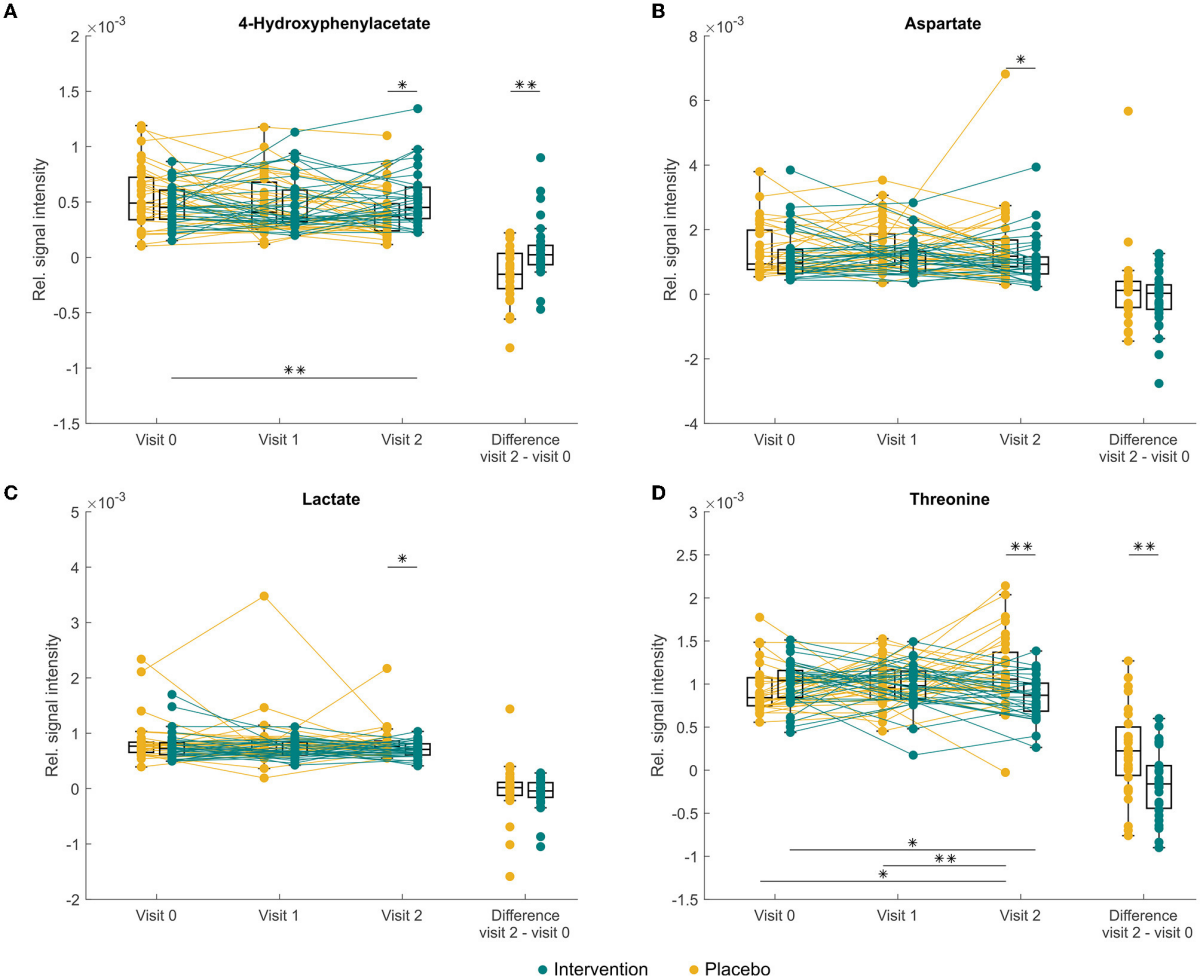
Changes in abundance of faecal **(A)** 4-hydroxyphenylacetate, **(B)** aspartate, **(C)** lactate, and threonine **(D)** in study participants either receiving a mixture of *Lactiplantibacillus plantarum* HEAL9 and *Lacticaseibacillus paracasei* 8700:2 (intervention) or placebo (placebo) at baseline (visit 0) and scheduled follow-up visits after 3 months (visit 1) and 6 months (visit 2). Asterisk indicates significant differences *p < 0.05, **p < 0.01 before applying the Benjamini–Hochberg correction, after the correction no significant changes were recorded.

In addition to the linear mixed-effects models, univariate statistics was performed with pairwise comparison for each visit separately. This approach has uncovered differences at the baseline between fumarate production, higher in the intervention group (*p* = 0.021) caused by an outlier in the treatment group. Thus, the two groups were considered equal in terms of metabolomic profile. The stool composition of donors under probiotic supplementation differed in lower ethanol (*p* = 0.017) and glycerol (*p* = 0.046) at 3 months when compared to the placebo group; nevertheless, a similar difference did not occur at 6 months visit. At 6 months, probiotic supplementation caused a significant increase of 4-hydroxyphenylacetate (*p* = 0.019, Figure 3A), while a decrease was reported in aspartate (*p* = 0.037, Figure 3B), lactate (*p* = 0.027, Figure 3C), and threonine (*p* = 0.001, Figure 3D) when compared to the baseline.

The pathway analysis showed depletion in six metabolic pathways; glycine, serine, and threonine metabolism (*p* = 0.013); cyanoamino acid metabolism (*p* = 0.024); methane metabolism (*p* = 0.025), pyruvate metabolism (*p* = 0.027); cysteine and methionine metabolism (*p* = 0.033); and nicotinate and nicotinamide metabolism (*p* = 0.049). The perturbed metabolic pathways in the faecal samples are shown in Supplementary Table 2 and Supplementary Figure 3.

### Associations between metabolome and microbiome

**Figure 4:**
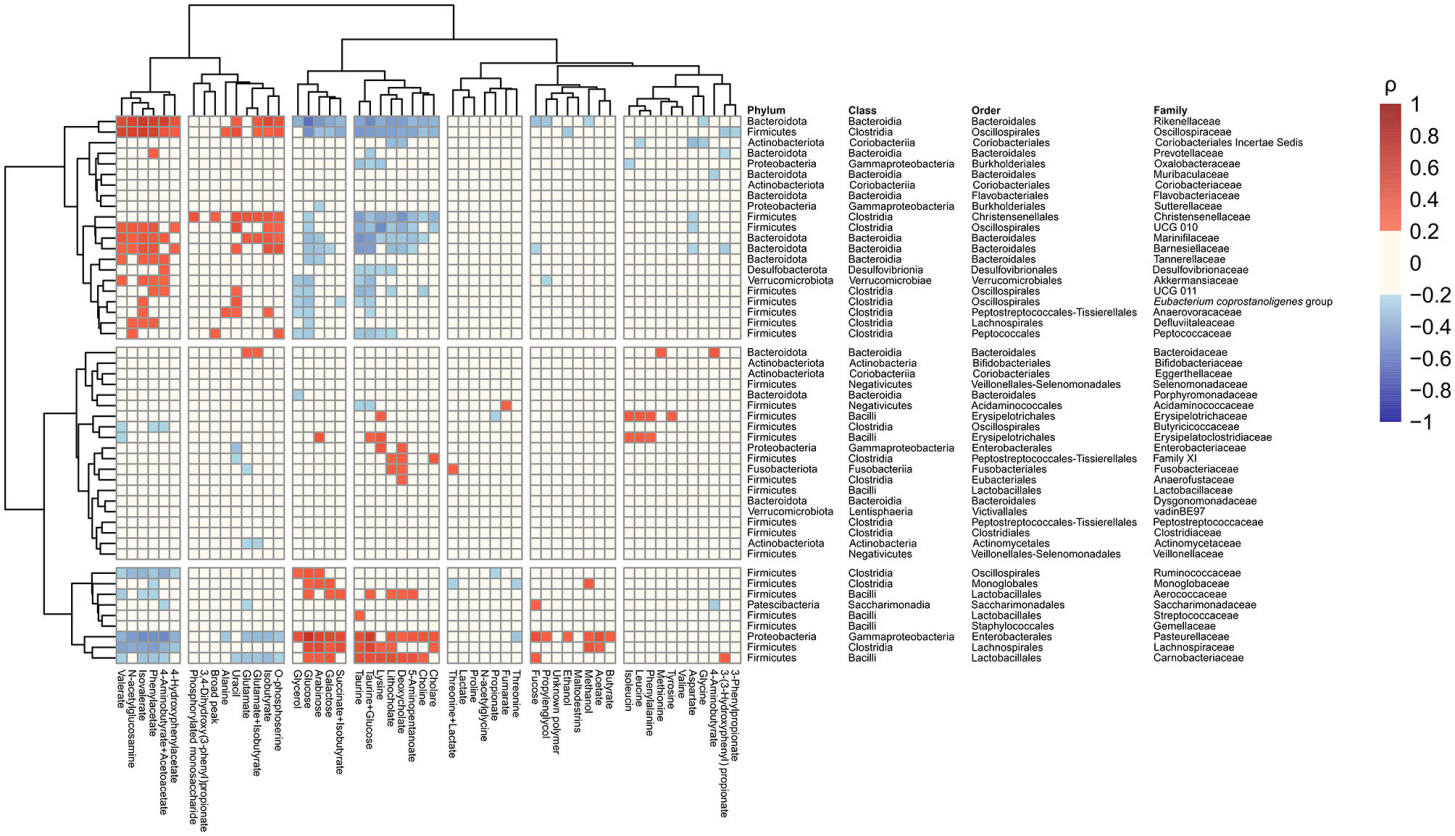
Correlations heatmap identifying associations between gut microbiota abundance at the family level and faecal metabolites in all study samples regardless of the visit and intervention.

**Figure 5:**
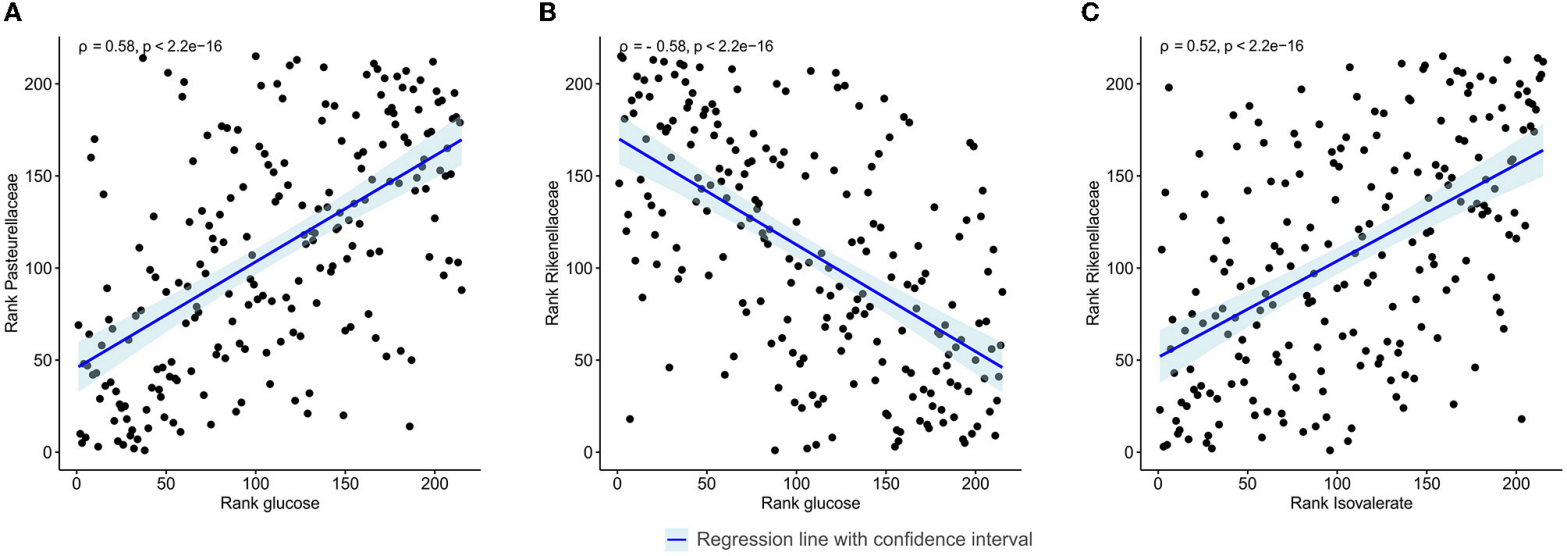
Spearman's rank correlation plot between gut microbiota abundance at the family level and faecal metabolites in all study samples regardless of the visit and intervention. **(A)** Linear regression between the family *Pasteurellaceae* and glucose. **(B)** Linear regression between the family *Rikenellaceae* and glucose. **(C)** Linear regression between the family *Rikenellaceae* and isovalerate. Correlations with ρ > |0.5| are shown.

The relative abundance of identified families and the concentration of the annotated metabolites showed that families *Pasteurellaceae, Monoglobaceae, Ruminococcaceae, Lachnospiraceae, Carnobacteriaceae*, and *Aerococcaceae* were positively associated with saccharolytic metabolites such as glucose and revealed negative correlation with proteolytic metabolites (Figure 4). Of particular interest was the inverse correlation between the family *Pasteurellaceae* and glucose (ρ = 0.58, *p* < 2.2 × 10^−16^; Figure 5A). The reverse was observed for families *Rikenellaceae, Oscillospiraceae, Christensenellaceae, Oscillospirales* family UCG-010, *Oscillospirales* family UCG-011, *Marinifilaceae, Barnesiellaceae, Akkermansiaceae, Eubacterium coprostanoligenes* group, *Anaerovoracaceae, Defluviitaleaceae, Peptococcaceae, Tannerellaceae*, and *Desulfovibrionaceae*, which were positively associated with an environment rich in proteolytic metabolites such as phenylacetate and negatively correlated with saccharolytic metabolites (Figure 4). An inverse correlation was observed between the family *Rikenellaceae* and glucose (ρ = −0.58, *p* < 2.2 × 10^−16^; Figure 5B). The abundance of *Rickenellaceae* diminished exponentially with increased glucose. On the other hand, the family *Rikenellaceae* was positively correlated with metabolite isovalerate (ρ = 0.52, *p* < 2.2 × 10^−16^; Figure 5C). The family *Lactobacillaceae* did not manifest any significant correlation.

## Discussion

The present study gives evidence that 6 months of intervention of *Lactiplantibacillus plantarum* HEAL9 and *Lacticaseibacillus paracasei* 8700:2 given to children with CD autoimmunity participating in a randomised, double-blinded placebo-controlled clinical trial has led to a significant decrease in threonine in the faecal metabolome and a significant rise in 4-hydroxyphenylacetate compared to the placebo group, as measured by ^1^H NMR and determined using linear mixed-effects models. There was also a tendency for other amino acids, such as valine, leucine, isoleucine, aspartate, methionine, and phenylalanine, together with fumarate, to decrease after the intervention. Their changes were borderline significant, though not after the adjustment for multiple comparisons.

All these metabolites are linked to protein breakdown and imply an effect on proteolytic fermentation in the gut. In the gastrointestinal tract, there is a delicate equilibrium between saccharolytic and proteolytic fermentation, and disturbances in this balance can be associated with various disease conditions (39, 40). Proteolytic fermentation, in general, leads to a protein, and peptides breakdown into amino acids, mainly resulting in the production of branched-chain fatty acids, polyamines, etc. (41), while saccharolytic fermentation degrades dietary fibre to simple carbohydrates to generate primarily SCFA and other organic acids (42). However, the suppression of the proteolytic pathway observed in our study was not translated into an increase in saccharolytic fermentation.

Proteolysis was previously shown to increase in disease states such as gut inflammation, where tissue damage occurs, and cells and cell exudates are released to the intestinal lumen and are subject to bacterial hydrolysis (43). In a study by Di Cagno et al. (44) which compared the metabolome of children with treated CD with healthy controls, a higher abundance of amino acids was found in the stool of CD children. Similar results were reported by De Angelis (45). In this light, our results may suggest a preventive nature of the probiotics and their potential ability to shape microbial metabolism towards a balanced state.

The decreased threonine concentration was the major change in the metabolome. Threonine synthesis by the human digestive system is limited, but it can be synthesised from glucose and aspartate by gut microbiota members (46). Additionally, we have seen a reduction in aspartate levels, and although we have not observed any alterations in glucose levels, the findings imply the regulation of this pathway. Furthermore, the pathway analysis confirmed an effect on threonine metabolism. In a previous study, which was based on this sample set examined using 16S rDNA sequencing, we noted an increase in abundance of the *Prevotella, Akkermansia, Streptococcus, and Bifidobacterium* genera (16). These genera lack a significant link to threonine in terms of its production, but *Akkermansia* is a prominent degrader of mucus (47). Mucus is a protective layer in the gut, and threonine is a highly abundant amino acid in the mucin protein core (48, 49). The *Akkermansia*, thus, may indirectly influence the threonine levels in the gut by degrading the mucus. However, increased levels of threonine would be expected based on this hypothesis, but a decrease was observed. This could be a sign of a healthier gut state as increased mucus degradation is associated with its physical disruption (50). Subsequent research on this finding is highly desirable.

4-Hydroxyphenylacetate, the only compound which increased after the intervention, is a colon microbial catabolite of tyrosine (51). This again suggests an effect on the proteolytic fermentation and breakdown of its metabolites. 4-Hydroxyphenylacetate itself may exhibit a biological effect in the gut, such as hepatoprotective and antioxidative properties (52). 4-Hydroxyphenylacetate does not seem to have a significant direct link to the altered genera in the same trial nor coeliac disease itself.

In addition to metabolome changes, the present study showed interesting correlations between microbiota and metabolites using all data regardless of the condition. Human gut microbial populations are characterised by several core taxa, including genera *Ruminococcae, Prevotella*, and *Bacteroides* (53). The first two genera are linked to the degradation of fibre or saccharolytic pathways, whereas *Bacteroides* are associated with proteolytic pathways. There are clear links with dietary intake as a plant-based diet is associated with higher levels of *Prevotella* and *Ruminococcus*, and meat consumption favours *Bacteroides* (54). Moreover, *Bacteroides* are increased in states of inflammation (55). In our correlation analysis, we detected strong correlations of the saccharolytic metabolites like glucose with families such as *Ruminococcaceae*, whereas other families such as *Rikenellaceae* showed correlations with amino acids and amino acid breakdown products. This clustering highly corresponds with the metabolic capacity of different taxa as mentioned above and shows the complementary nature of the two methods.

## Conclusion

The combination of *Lactiplantibacillus plantarum* HEAL9 and *Lacticaseibacillus paracasei* 8700:2 showed marginal, though significant changes in the levels of numerous stool metabolites in children genetically predisposed to CD after 6 months of intervention, mainly consisting of differences in amino acids profiles, indicating a shift towards known healthier metabolic patterns. The observed reduction in threonine levels is also worth emphasising, as threonine is an amino acid closely associated with gut mucus. The metabolic pathway related to threonine appears to be impacted by probiotic the intervention.

## Data availability statement

The datasets presented in this study can be found in online repositories. The names of the repository/repositories and accession number(s) can be found below: https://www.ncbi.nlm.nih.gov/, PRJNA732664.

## Ethics statement

The studies involving human participants were reviewed and approved by Ethics Committee of the Medical Faculty, Lund University (Dnr 2011/335; Dnr 2021-04470). Written informed consent to participate in this study was provided by the participants' legal guardian/next of kin.

## Author contributions

EJ drafted the manuscript, interpreted the data, and completed all subsequent revisions until submission. CAA coordinated the collection of the samples and data collection from study participants. EJ and JH performed the metabolomic analysis. OC performed the microbiological analysis and reviewed and revised the manuscript. AMB carried out the statistical analysis. DA was the principal investigator for the study and responsible for study design. DA and JH conceptualised the study, advised in the presentation of analysis results, interpreted the data, reviewed and revised the manuscript, and critically evaluated for important intellectual content. All authors contributed to the article and approved the submitted version.
