# Supplementary material for: Effects of Lactiplantibacillus plantarum and Lacticaseibacillus paracasei supplementation on the faecal metabolome in children with coeliac disease autoimmunity: a randomised, double-blinded placebo-controlled clinical trial
Source: Front Nutr. 2023 Jul 6;10:1183963. doi: 10.3389/fnut.2023.1183963 (PMC10359497; doi:10.3389/fnut.2023.1183963)
Supplement: Supplementary file 1 [file Data_Sheet_1.docx]

Supplementary Material

Effects of *Lactiplantibacillus* *plantarum* and *Lacticaseibacillus paracasei* supplementation on the faecal metabolome in children with coeliac disease autoimmunity: a randomised, double-blinded placebo-controlled clinical trial

**Eliska Jenickova, Carin Andren Aronsson, Anna Mascellani Bergo, Ondrej Cinek, Jaroslav Havlik, Daniel Agardh^*^, and** **the HEDIMED Investigator Group.**

*** Correspondence:**Daniel Agardh,

daniel.agardh@med.lu.se

**Supplementary Tables:**

**Supplementary Table 1:** List of buckets included in the study covering spectrum region between δ_H_ 0.5–9.0 ppm (excluding the residual water region, δ_H_ 5.1–4.6 ppm) defined based on previously recorded features. Buckets may contain either annotated or unknown peaks. Additionally, changes in the area of the buckets in study participants either receiving a mixture of *Lactiplantibacillus* *plantarum* HEAL9 and *Lacticaseibacillus* *paracasei* 8700:2 (intvn) or placebo (plcbo) at baseline (v0) and scheduled follow-up visits after three months (v1) and six months (v2) are shown.

| **Bucket name** | **Bucket range**  (ppm)  start  end | **Plcbo**  **v0 vs v1** | **Plcbo**  **v1 vs v2** | **Plcbo**  **v0 vs v2** | **Intvn.**  **v0 vs v1** | **Intvn**  **v0 vs v1** | **Intvn**  **v0 vs v1** | **v0**  **Plcb vs intvn** | **v1**  **Plcb vs intvn** | **v2**  **Plcb vs intvn** | **Difference**  **v2 - v0**  **Plcb vs intvn** |
| --- | --- | --- | --- | --- | --- | --- | --- | --- | --- | --- | --- |
| 3-(3-hydroxyphenyl) propionate | 6.7380  6.7560 | 0.468 | 0.299 | 0.088 | 0.217 | 0.264 | 0.739 | 0.125 | 0.414 | 0.285 | 0.125 |
| 3-(3-hydroxyphenyl) propionate | 6.7560  6.7730 | 0.505 | 0.624 | 0.153 | 0.281 | 0.481 | 0.468 | 0.076 | 0.612 | 0.318 | 0.172 |
| 3,4-dihydroxy(3-phenyl) propionate | 6.7100  6.7220 | 0.217 | 0.078 | 0.378 | 0.518 | 0.845 | 0.518 | 0.944 | 0.439 | 0.186 | 0.422 |
| 3,4-dihydroxy(3-phenyl) propionate | 6.7270  6.7380 | 0.829 | 0.299 | 0.337 | 0.481 | 0.891 | 0.891 | 0.833 | 0.499 | 0.383 | 0.642 |
| 3-Phenylpropionate | 7.2380  7.2490 | 0.264 | 0.422 | 0.158 | **0.006** | **0.033** | 0.860 | 0.052 | 0.353 | 1.000 | 0.353 |
| 4-Aminobutyrate | 2.2700  2.2770 | 0.164 | 0.337 | 0.652 | 0.456 | 0.481 | 0.327 | 0.866 | 0.866 | 0.147 | 0.390 |
| 4-Aminobutyrate + Acetoacetate | 2.2560  2.2640 | 0.367 | 0.505 | 0.984 | 0.367 | 0.308 | 0.065 | 0.554 | 0.481 | 0.097 | 0.168 |
| 4-Hydroxyphenylacetate | 6.8690  6.8840 | 0.240 | 0.122 | **0.003** | 0.814 | 0.183 | 0.240 | 0.318 | 0.573 | **0.019** | **0.003** |
| 4-Hydroxyphenylacetate | 7.1570  7.1700 | 0.337 | 0.829 | 0.224 | 0.433 | 0.610 | 0.389 | 0.226 | 0.877 | 0.272 | 0.168 |
| 5-Aminopentanoate | 1.6420  1.6520 | 0.695 | 0.984 | 0.610 | 0.583 | 0.666 | 0.666 | 0.278 | 0.844 | 0.877 | 0.499 |
| Acetate | 1.9100  1.9330 | 0.784 | 0.410 | 0.710 | 0.724 | 0.065 | 0.092 | 0.526 | 0.822 | 0.088 | 0.177 |
| Alanine | 1.4680  1.4820 | 0.122 | 0.906 | 0.170 | 0.814 | 0.299 | 0.570 | 0.297 | 0.767 | 0.499 | 0.231 |
| Arabinose | 4.5120  4.5190 | 0.597 | 0.638 | 0.583 | 0.299 | 0.153 | 0.754 | 0.456 | 0.910 | 0.473 | 0.464 |
| Aspartate | 2.7030  2.7160 | 0.468 | 0.337 | 0.938 | 0.456 | 0.597 | 0.799 | 0.331 | 0.168 | 0.118 | 0.888 |
| Aspartate | 2.7880  2.8100 | 0.308 | 0.557 | 0.922 | 0.891 | 0.493 | 0.544 | 0.398 | 0.181 | 0.037 | 0.622 |
| Butyrate | 1.5630  1.6050 | 0.232 | 0.695 | 0.318 | 0.570 | 0.814 | 0.378 | 0.767 | 0.966 | 0.243 | 0.231 |
| Choline | 3.2010  3.2100 | 0.638 | 0.256 | 0.104 | 0.597 | 0.337 | 0.638 | 0.593 | 0.383 | 0.622 | 0.481 |
| Ethanol | 1.1990  1.2070 | 0.468 | 0.652 | 0.922 | 0.057 | 0.327 | 0.468 | 0.564 | **0.017** | 0.318 | 0.693 |
| Fucose | 5.2030  5.2110 | 0.357 | 0.347 | 0.518 | 0.610 | 0.681 | 0.724 | 0.725 | 0.632 | 0.642 | 0.602 |
| Fucose | 1.2410  1.2470 | 0.232 | 0.057 | 0.953 | 0.240 | 0.126 | 0.875 | 0.714 | 1.000 | 0.642 | 0.789 |
| Fucose | 1.2550  1.2600 | 0.337 | 0.117 | 0.308 | 0.224 | 0.126 | 0.505 | 0.554 | 0.888 | 0.673 | 0.800 |
| Fucose | 5.2110  5.2190 | 0.232 | 0.170 | 0.378 | 0.357 | 0.327 | 0.845 | 0.767 | 0.536 | 0.757 | 0.583 |
| Fumarate | 6.5180  6.5320 | 0.170 | 0.583 | **0.033** | 0.724 | 0.845 | 0.695 | **0.021** | 0.159 | 0.447 | 0.221 |
| Galactose | 4.5950  4.6050 | 0.422 | 0.906 | 0.784 | 0.445 | 0.754 | 0.203 | 0.526 | 0.481 | 0.866 | 0.414 |
| Galactose | 5.2670  5.2830 | 0.638 | 0.610 | 0.367 | **0.014** | 0.203 | 0.272 | 0.147 | 0.921 | 0.877 | 0.172 |
| Glucose | 3.2280  3.2390 | 0.531 | 0.445 | 0.799 | 0.055 | 0.281 | 0.224 | 0.360 | 0.844 | 0.663 | 0.517 |
| Glucose | 3.4930  3.5050 | 0.784 | 0.681 | 0.969 | **0.033** | 0.142 | 0.081 | 0.243 | 0.767 | 0.593 | 0.272 |
| Glucose | 5.2290  5.2520 | 0.652 | 0.389 | 0.984 | **0.046** | 0.131 | 0.100 | 0.260 | 0.944 | 0.789 | 0.243 |
| Glutamate | 2.3360  2.3470 | **0.031** | 0.531 | 0.299 | 0.557 | 0.389 | 0.183 | 0.172 | 0.910 | 0.125 | 0.899 |
| Glutamate + Isobutyrate | 2.3700  2.3820 | 0.040 | 0.357 | 0.666 | 0.739 | 0.410 | 0.248 | 0.291 | 0.683 | 0.109 | 0.536 |
| Glycerol | 3.5260  3.5310 | 0.583 | 0.610 | 0.845 | 0.033 | 0.078 | 0.196 | 0.439 | 0.390 | 0.899 | 0.338 |
| Glycerol | 3.5460  3.5520 | 0.347 | 0.829 | 0.378 | **0.036** | 0.078 | 0.739 | 0.714 | **0.046** | 0.704 | 0.652 |
| Glycine | 3.5630  3.5710 | 0.136 | 0.570 | **0.048** | 0.290 | 0.583 | 0.272 | 0.324 | 0.272 | 0.237 | 0.063 |
| Isobutyrate | 2.3860  2.3960 | 0.433 | 0.610 | 0.570 | 0.557 | 0.953 | 0.357 | 0.573 | 0.612 | 0.833 | 0.767 |
| Isoleucine | 1.0150  1.0255 | 0.131 | 0.845 | 0.088 | 0.984 | 0.378 | 0.493 | 0.177 | 0.499 | 0.622 | 0.109 |
| Isovalerate | 0.9180  0.9240 | 0.769 | 0.681 | 0.505 | 0.557 | 0.557 | 0.327 | 0.368 | 0.430 | 0.735 | 0.285 |
| Isovalerate | 0.9040  0.9100 | 0.399 | 0.724 | 0.378 | 0.570 | 0.399 | 0.176 | 0.311 | 0.508 | 0.278 | 0.151 |
| Lactate | 4.0990  4.1050 | 0.624 | 0.337 | 0.695 | 0.367 | 0.597 | 0.422 | 0.368 | 0.447 | **0.027** | 0.439 |
| Lactate | 4.1410  4.1460 | 0.875 | 0.493 | 0.210 | 0.557 | 0.906 | 0.456 | 0.151 | 0.109 | 0.205 | 0.499 |
| Lactate | 4.1250  4.1310 | 0.666 | 0.666 | 0.518 | 0.117 | 0.597 | 0.096 | 0.422 | 0.078 | 0.030 | 0.398 |
| Leucine | 0.9590  0.9790 | 0.081 | 0.739 | 0.088 | 0.814 | 0.112 | 0.153 | 0.200 | 0.910 | 0.360 | 0.038 |
| Lysine | 3.0270  3.0370 | 0.953 | 0.799 | 0.570 | 0.891 | 0.445 | 0.327 | 0.910 | 0.612 | 0.602 | 0.642 |
| Methanol | 3.3610  3.3710 | 0.456 | 0.122 | 0.814 | 0.829 | 0.845 | 0.248 | 0.855 | 0.291 | 0.159 | 0.304 |
| Methionine | 2.6420  2.6510 | **0.040** | 0.829 | 0.042 | 0.610 | 0.337 | 0.829 | 0.254 | 0.899 | 0.346 | 0.115 |
| Methionine | 2.1370  2.1440 | 0.232 | 0.906 | 0.203 | 0.860 | 0.969 | 0.845 | 0.200 | 0.481 | 0.735 | 0.383 |
| *N*-acetylglucosamine | 2.0510  2.0560 | 0.799 | 0.969 | 0.681 | 0.422 | 0.597 | 0.308 | 0.272 | 0.481 | 0.800 | 0.291 |
| *N*-acetylglycine | 2.0450  2.0510 | 0.367 | 0.769 | 0.860 | 0.724 | 0.126 | 0.092 | 0.767 | 0.652 | 0.094 | 0.210 |
| *O*-phosphoserine | 4.1620  4.2130 | 0.652 | 0.695 | 0.610 | 0.610 | 0.544 | 0.203 | 0.195 | 0.583 | 0.086 | 0.231 |
| Phenylacetate | 3.5390  3.5450 | 0.210 | 0.938 | 0.456 | 0.347 | 0.164 | 0.131 | 0.067 | 0.439 | 0.899 | 0.069 |
| Phenylacetate | 7.3040  7.3190 | 0.739 | 0.860 | 0.638 | 0.290 | 0.158 | 0.096 | 0.346 | 0.767 | 0.375 | 0.139 |
| Phenylacetate | 7.3690  7.3770 | 0.681 | 0.799 | 0.953 | 0.203 | 0.544 | 0.203 | 0.683 | 0.989 | 0.490 | 0.375 |
| Phenylacetate | 7.4010  7.4070 | 0.845 | 0.739 | 0.784 | 0.272 | 0.724 | 0.389 | 0.822 | 0.602 | 0.693 | 0.439 |
| Phenylalanine | 7.3350  7.3500 | 0.117 | 0.695 | 0.505 | 0.969 | 0.092 | 0.217 | 0.338 | 0.573 | 0.439 | 0.168 |
| Phosphorylated monosaccharide (tentatively) | 5.6090  5.6160 | 0.038 | 0.891 | 0.085 | 0.468 | 0.724 | 0.953 | 0.231 | 0.955 | 0.746 | 0.272 |
| Proline | 3.3490  3.3600 | 0.308 | 0.754 | 0.290 | **0.023** | 0.505 | 0.122 | 0.652 | 0.076 | 0.065 | 0.078 |
| Proline | 4.1480  4.1560 | 0.739 | 0.597 | 0.389 | 0.518 | 0.681 | 0.136 | 0.318 | 0.091 | 0.069 | 0.735 |
| Propionate | 2.1910  2.1990 | 0.652 | 0.557 | 0.829 | 0.799 | 0.164 | 0.096 | 0.966 | 0.564 | 0.097 | 0.210 |
| Propionate | 2.2070  2.2140 | 0.845 | 0.652 | 0.681 | 0.953 | 0.232 | 0.100 | 0.746 | 0.899 | 0.094 | 0.221 |
| Propylene glycol | 3.6360  3.7580 | 0.860 | 0.681 | 0.308 | 0.570 | 0.799 | 0.875 | 0.554 | 0.383 | 0.725 | 0.517 |
| Succinate + Isobutyrate | 2.4010  2.4250 | 0.953 | 0.845 | 0.784 | 1.000 | 0.410 | 0.875 | 0.989 | 0.231 | 0.989 | 0.789 |
| Taurine | 3.2770  3.2850 | 0.210 | 0.597 | 0.196 | 0.100 | 0.799 | 0.217 | 0.456 | 0.473 | 0.456 | 0.955 |
| Taurine + Glucose | 3.2630  3.2740 | 0.799 | 0.799 | 0.433 | 0.022 | 0.272 | 0.272 | 0.083 | 0.714 | 0.324 | 0.735 |
| Threonine | 4.2540  4.2670 | 0.153 | 0.136 | **0.025** | 0.583 | **0.009** | 0.040 | 0.078 | 0.877 | **0.001** | **0.004** |
| Threonine | 4.2410  4.2540 | 0.505 | 0.318 | 0.318 | 0.681 | 0.158 | 0.337 | 0.554 | 0.564 | **0.003** | 0.143 |
| Threonine | 4.2670  4.2750 | 0.248 | 0.057 | **0.020** | 0.681 | 0.126 | 0.112 | 0.115 | 0.683 | **0.001** | **0.003** |
| Threonine + Lactate | 1.3220  1.3340 | 0.085 | 0.357 | 0.557 | 0.695 | 0.153 | 0.196 | 0.237 | 0.989 | 0.406 | 0.139 |
| Tyrosine | 6.8880  6.9040 | 0.108 | 0.829 | 0.739 | 0.445 | 0.337 | 0.922 | 0.177 | 0.833 | 0.517 | 0.789 |
| Uracil | 5.8050  5.8210 | **0.040** | 0.906 | 0.126 | 0.829 | 0.784 | 0.953 | 0.304 | 0.622 | 0.338 | 0.260 |
| Valerate | 1.5060  1.5210 | 0.906 | 0.739 | 0.389 | 0.399 | 0.953 | 0.117 | 0.545 | 0.778 | 0.210 | 0.086 |
| Valerate | 2.1680  2.1730 | 0.610 | 0.445 | 0.481 | 0.724 | 0.357 | 0.189 | 0.811 | 0.663 | 0.065 | 0.159 |
| Valine | 1.0330  1.0410 | 0.096 | 0.906 | 0.050 | 0.875 | 0.638 | 0.784 | 0.125 | 0.499 | 0.789 | 0.118 |
| Valine | 0.9940  1.0030 | 0.060 | 0.906 | 0.046 | 0.984 | 0.240 | 0.481 | 0.163 | 0.473 | 0.390 | 0.076 |
| Bucket1 | 0.650  0.690 | 0.860 | 0.445 | 0.378 | 0.044 | 0.399 | **0.021** | 0.811 | 0.714 | 0.139 | 0.115 |
| Bucket2 | 0.690  0.747 | 0.610 | 0.505 | 0.117 | 0.445 | 0.071 | 0.085 | 0.490 | 0.811 | 0.210 | 0.473 |
| Bucket3 | 0.747  0.761 | 0.030 | 0.203 | 0.984 | 0.170 | 0.666 | 0.088 | 0.778 | 0.877 | **0.007** | 0.118 |
| Bucket4 | 0.800  0.817 | 0.814 | 0.799 | 0.891 | 0.153 | 0.078 | 0.378 | 0.554 | 0.554 | 0.693 | 0.725 |
| Bucket5 | 0.835  0.843 | 0.557 | 0.891 | 0.281 | 0.158 | 0.875 | 0.232 | 0.254 | 0.910 | 0.517 | 0.083 |
| Bucket6 | 0.843  0.850 | 0.378 | 0.938 | 0.378 | 0.272 | 0.544 | 0.189 | 0.210 | 0.789 | 0.612 | 0.132 |
| Bucket7 | 0.850  0.858 | 0.638 | 0.290 | 0.518 | 0.158 | 0.248 | 0.085 | 0.447 | 0.877 | 0.147 | 0.100 |
| Bucket8 | 0.857  0.862 | 0.681 | 0.922 | 0.710 | 0.196 | 0.481 | 0.117 | 0.368 | 0.855 | 0.517 | 0.205 |
| Bucket9 | 0.862  0.867 | 0.953 | 0.739 | 0.860 | 0.799 | 0.422 | 0.299 | 0.855 | 0.899 | 0.866 | 0.593 |
| Bucket10 | 0.872  0.881 | 0.769 | 0.969 | 0.531 | 0.984 | 0.224 | 0.170 | 0.430 | 0.757 | 0.159 | 0.237 |
| Bucket11 | 0.888  0.895 | 0.456 | 0.624 | 0.468 | 0.638 | 0.196 | 0.210 | 0.406 | 0.517 | 0.086 | 0.200 |
| Bucket12 | 1.103  1.114 | 0.203 | 0.136 | 0.389 | 0.075 | 0.142 | 0.695 | 0.833 | 0.910 | 0.822 | 0.346 |
| Bucket13 | 1.117  1.122 | 0.170 | 0.493 | 0.597 | 0.210 | 0.048 | 0.203 | 0.877 | 0.778 | 0.115 | 0.205 |
| Bucket14 | 1.177  1.185 | 0.695 | 0.248 | 0.456 | 0.422 | 0.695 | 0.610 | 0.331 | 0.642 | 0.573 | 0.414 |
| Bucket15 | 1.263  1.318 | 0.624 | 0.666 | 0.739 | 0.456 | 0.784 | 0.433 | 0.899 | 0.360 | 0.554 | 0.390 |
| Bucket16 | 1.482  1.486 | 0.290 | 0.624 | 0.518 | 0.891 | 0.875 | 0.906 | 0.714 | 0.693 | 0.622 | 0.714 |
| Bucket17 | 1.497  1.504 | 0.378 | 0.969 | 0.922 | 0.681 | 0.984 | 0.281 | 0.833 | 0.877 | 0.877 | 0.757 |
| Bucket18 | 1.528  1.534 | 0.624 | 0.505 | 0.196 | 0.860 | 0.468 | 0.203 | 0.318 | 0.866 | 0.136 | 0.100 |
| Bucket19 | 1.543  1.548 | 0.666 | 0.357 | 0.224 | 0.875 | 0.445 | 0.272 | 0.473 | 0.899 | 0.094 | 0.118 |
| Bucket20 | 1.612  1.622 | 0.610 | 0.624 | 0.969 | 0.984 | 0.938 | 0.984 | 0.921 | 0.933 | 0.966 | 0.910 |
| Bucket21 | 1.792  1.799 | 0.318 | 0.829 | 0.367 | 0.264 | 0.117 | 0.217 | 0.866 | 0.683 | 0.094 | 0.168 |
| Bucket22 | 1.867  1.876 | 0.224 | 0.984 | 0.422 | 0.433 | 0.845 | 0.337 | 0.866 | 0.693 | 0.536 | 0.866 |
| Bucket23 | 1.887  1.909 | 0.969 | 0.860 | 0.570 | 0.327 | 0.610 | 0.327 | 0.933 | 0.536 | 0.855 | 0.704 |
| Bucket24 | 1.945  1.952 | 0.829 | 0.583 | 0.104 | **0.005** | **0.046** | 0.308 | 0.136 | 0.291 | 0.517 | 0.086 |
| Bucket25 | 1.952  1.964 | 0.906 | 0.481 | 0.096 | **0.002** | 0.065 | 0.096 | 0.094 | 0.190 | 0.172 | **0.027** |
| Bucket26 | 1.979  1.991 | 0.652 | 0.710 | 0.164 | 0.531 | 0.060 | 0.481 | 0.278 | 0.673 | 0.151 | 0.573 |
| Bucket27 | 1.991  1.998 | 0.224 | 0.769 | 0.088 | 0.695 | **0.018** | 0.170 | 0.278 | 0.966 | 0.221 | 0.693 |
| Bucket28 | 1.998  2.007 | 0.814 | 0.638 | 0.468 | 0.445 | 0.085 | 0.034 | 0.622 | 0.855 | 0.168 | 0.266 |
| Bucket29 | 2.007  2.018 | 0.399 | 0.153 | 0.953 | 0.638 | 0.272 | 0.518 | 0.602 | 0.285 | 0.272 | 0.481 |
| Bucket30 | 2.056  2.063 | 0.969 | 0.104 | 0.063 | **0.044** | 0.337 | 0.493 | 0.243 | 0.481 | 0.375 | 0.100 |
| Bucket31 | 2.235  2.242 | 0.891 | 0.378 | 0.481 | 0.724 | 0.681 | 0.754 | 0.508 | 0.855 | 0.375 | 0.622 |
| Bucket32 | 2.279  2.283 | **0.024** | 0.493 | 0.108 | 0.281 | 0.468 | 0.906 | 0.447 | 0.735 | 0.921 | 0.311 |
| Bucket33 | 2.298  2.305 | 0.164 | 0.724 | 0.357 | 0.799 | 0.389 | 0.938 | 0.673 | 0.899 | 0.632 | 0.517 |
| Bucket34 | 2.327  2.336 | 0.183 | 0.357 | **0.010** | 0.357 | 0.256 | 0.493 | 0.297 | 0.966 | 0.955 | 0.490 |
| Bucket35 | 2.442  2.456 | 0.829 | 0.493 | 0.122 | 0.638 | 0.769 | 0.481 | 0.933 | 0.855 | 0.955 | 0.439 |
| Bucket36 | 2.471  2.484 | 0.318 | 0.456 | 0.060 | 0.724 | 0.468 | 0.906 | 0.508 | 0.683 | 0.583 | 0.177 |
| Bucket37 | 2.504  2.510 | 0.240 | 0.666 | 0.610 | 0.906 | 0.860 | 0.681 | 0.490 | 0.190 | 0.966 | 0.955 |
| Bucket38 | 2.510  2.515 | 0.153 | 0.891 | 0.078 | 0.710 | 0.367 | 0.232 | 0.612 | 0.632 | 0.291 | 0.844 |
| Bucket39 | 2.521  2.529 | 0.040 | 0.170 | 0.433 | 0.367 | 0.399 | 0.799 | 0.554 | **0.005** | 0.414 | 0.714 |
| Bucket40 | 2.570  2.578 | 0.081 | 0.050 | 0.891 | 0.906 | 0.158 | 0.183 | 0.383 | 0.439 | **0.030** | 0.406 |
| Bucket41 | 2.591  2.605 | 0.183 | 0.378 | 0.891 | **0.016** | 0.347 | **0.005** | 0.767 | 0.545 | **0.019** | **0.044** |
| Bucket42 | 2.605  2.612 | 0.367 | 0.666 | 0.493 | 0.624 | 0.108 | 0.057 | 0.933 | 0.642 | 0.237 | 0.398 |
| Bucket43 | 2.716  2.726 | 0.024 | 0.100 | 0.544 | 0.318 | 0.681 | 0.203 | 0.226 | 0.061 | 0.663 | 0.693 |
| Bucket44 | 2.726  2.731 | 0.378 | 0.433 | 0.681 | 0.875 | 0.829 | 0.969 | 0.439 | 0.383 | 0.683 | 0.704 |
| Bucket45 | 2.749  2.755 | 0.433 | 0.710 | 0.570 | **0.002** | 0.071 | 0.318 | 0.168 | 0.375 | 0.593 | 0.811 |
| Bucket46 | 2.755  2.763 | 0.505 | 0.337 | 0.136 | 1.000 | 0.217 | 0.153 | 0.554 | 0.966 | 0.128 | **0.049** |
| Bucket47 | 2.810  2.821 | 0.433 | 0.433 | 0.096 | 0.695 | 0.147 | 0.299 | 0.248 | 0.226 | 0.406 | 0.593 |
| Bucket48 | 2.821  2.829 | 0.570 | 0.784 | 0.176 | 0.399 | 0.078 | 0.829 | 0.105 | 0.704 | 0.554 | 0.360 |
| Bucket49 | 2.840  2.847 | 0.272 | 0.984 | 0.240 | 0.891 | **0.013** | 0.327 | 0.078 | 0.526 | 0.353 | 0.800 |
| Bucket50 | 2.860  2.867 | 0.308 | 0.695 | 0.399 | 0.126 | 0.240 | 0.829 | 0.683 | 0.353 | 0.866 | 0.346 |
| Bucket51 | 2.877  2.887 | 0.337 | 0.754 | 0.399 | 0.531 | **0.024** | 0.170 | 0.725 | 0.331 | 0.406 | 0.693 |
| Bucket52 | 2.887  2.892 | 0.445 | 0.357 | 1.000 | 0.060 | 0.044 | 0.531 | 0.564 | 0.714 | 0.331 | 0.704 |
| Bucket53 | 2.892  2.903 | 0.969 | 0.754 | 0.557 | 0.544 | **0.040** | 0.126 | 0.564 | 1.000 | 0.044 | 0.118 |
| Bucket54 | 2.907  2.916 | 0.891 | 0.389 | 0.570 | 0.433 | **0.016** | 0.142 | 0.704 | 0.508 | 0.059 | 0.115 |
| Bucket55 | 2.925  2.935 | 0.399 | 0.248 | 0.422 | **0.042** | 0.126 | 0.481 | 0.767 | 0.833 | 0.811 | 0.877 |
| Bucket56 | 2.935  2.941 | 0.327 | 1.000 | 0.203 | 0.583 | 0.583 | 0.183 | 0.473 | 0.297 | 0.151 | 0.052 |
| Bucket57 | 2.941  2.944 | 0.088 | **0.046** | 0.829 | 0.695 | 0.784 | 0.652 | 0.311 | 0.318 | 0.632 | 0.612 |
| Bucket58 | 2.944  2.950 | 0.518 | 0.389 | 0.681 | 0.256 | 0.666 | 0.176 | 0.844 | 0.414 | 0.593 | 0.652 |
| Bucket59 | 2.975  2.981 | 0.845 | 0.666 | 0.493 | 0.638 | 0.142 | 0.071 | 0.933 | 0.800 | 0.205 | 0.065 |
| Bucket60 | 2.981  2.990 | 0.784 | 0.652 | 0.739 | 0.481 | 0.347 | 0.081 | 0.989 | 0.593 | 0.490 | 0.297 |
| Bucket61 | 2.990  3.013 | 0.922 | 0.938 | 0.754 | 0.624 | 0.518 | 0.224 | 0.422 | 0.499 | 0.375 | 0.526 |
| Bucket62 | 3.013  3.025 | 0.922 | 0.493 | 0.984 | 0.739 | 0.557 | 0.493 | 0.673 | 0.800 | 0.439 | 0.704 |
| Bucket63 | 3.025  3.041 | 0.695 | 0.493 | 0.938 | 0.724 | 0.724 | 0.318 | 0.789 | 0.833 | 0.390 | 0.583 |
| Bucket64 | 3.041  3.055 | 0.357 | 0.769 | 0.240 | 0.799 | 0.829 | 0.891 | 0.767 | 0.944 | 0.632 | 0.473 |
| Bucket65 | 3.055  3.071 | 0.433 | 0.597 | 0.217 | 0.597 | 0.799 | 0.814 | 0.545 | 0.324 | 0.132 | 0.368 |
| Bucket66 | 3.071  3.085 | 0.710 | 0.136 | 0.248 | 0.456 | 0.357 | 0.505 | 0.473 | 0.622 | 0.704 | 0.767 |
| Bucket67 | 3.103  3.117 | 0.695 | 0.724 | 0.938 | 0.906 | **0.007** | 0.057 | 0.778 | 0.464 | 0.297 | 0.139 |
| Bucket68 | 3.128  3.141 | 0.845 | 0.754 | 0.290 | 0.814 | 0.100 | 0.389 | 0.490 | 0.159 | **0.049** | 0.260 |
| Bucket69 | 3.150  3.163 | 0.088 | 0.248 | **0.005** | 0.112 | **0.023** | 0.875 | 0.789 | 0.536 | 0.291 | 0.049 |
| Bucket70 | 3.194  3.200 | 0.272 | 0.085 | **0.011** | **0.044** | 0.136 | 0.570 | 0.844 | 0.481 | 0.490 | 0.139 |
| Bucket71 | 3.242  3.247 | 0.922 | 0.183 | 0.078 | 0.224 | 0.681 | 0.456 | 1.000 | 0.642 | 0.683 | 0.593 |
| Bucket72 | 3.256  3.260 | 0.984 | 0.422 | 0.158 | 0.142 | 0.784 | 0.493 | 0.855 | 0.822 | 0.888 | 0.622 |
| Bucket73 | 3.289  3.300 | 0.481 | 0.570 | 0.953 | **0.024** | 0.183 | 0.953 | 0.473 | 0.186 | 0.602 | 0.955 |
| Bucket74 | 3.300  3.313 | 0.695 | 0.695 | 0.906 | 0.210 | 0.347 | 0.814 | 0.899 | 0.430 | 0.767 | 0.866 |
| Bucket75 | 3.313  3.321 | 0.399 | 0.557 | 0.906 | 0.068 | 0.531 | 0.389 | 0.673 | 0.054 | 0.800 | 0.414 |
| Bucket76 | 3.321  3.327 | 0.433 | 0.505 | 0.754 | 0.057 | 0.104 | 0.784 | 0.978 | 0.186 | 0.414 | 0.693 |
| Bucket77 | 3.327  3.332 | 0.224 | 0.347 | 0.638 | 0.050 | 0.290 | 0.170 | 0.536 | **0.047** | 0.499 | 0.490 |
| Bucket78 | 3.332  3.336 | 0.875 | 0.505 | 0.638 | 0.570 | 0.196 | 0.210 | 0.136 | **0.027** | 0.642 | 0.143 |
| Bucket79 | 3.336  3.349 | 0.922 | 0.610 | 0.597 | 0.327 | 0.232 | 0.845 | 0.490 | 0.069 | 0.583 | 0.800 |
| Bucket80 | 3.602  3.609 | 0.624 | 0.906 | 0.481 | 0.984 | 0.984 | 0.984 | 0.210 | 0.573 | 0.725 | 0.622 |
| Bucket81 | 3.636  3.665 | 0.953 | 0.984 | 0.445 | 0.176 | 0.232 | 0.544 | 0.933 | 0.065 | 0.231 | 0.311 |
| Bucket82 | 3.655  3.660 | 0.799 | 0.829 | 0.610 | 0.147 | 0.456 | 0.240 | 0.422 | 0.508 | 0.612 | 0.725 |
| Bucket83 | 3.670  3.673 | 0.597 | 0.544 | 0.769 | 0.081 | 0.695 | 0.210 | 0.693 | 0.526 | 0.564 | 0.536 |
| Bucket84 | 3.829  3.835 | 0.610 | 0.784 | 0.445 | 0.164 | 0.799 | 0.203 | 0.855 | 0.237 | 0.221 | 0.757 |
| Bucket85 | 3.835  3.845 | 0.784 | 0.814 | 0.468 | 0.092 | 0.891 | 0.189 | 0.921 | 0.186 | 0.195 | 0.683 |
| Bucket86 | 3.845  3.855 | 0.953 | 0.570 | 0.389 | 0.248 | 0.481 | 0.281 | 0.683 | 0.272 | 0.136 | 0.899 |
| Bucket87 | 3.855  3.868 | 0.468 | 0.544 | 0.063 | 0.153 | 0.399 | 0.158 | 0.311 | 0.266 | 0.151 | 0.735 |
| Bucket88 | 3.874  3.882 | 0.624 | 0.938 | 0.100 | 0.081 | 0.638 | 0.126 | 0.439 | 0.390 | 0.094 | 0.944 |
| Bucket89 | 3.899  3.905 | 0.092 | 0.610 | 0.112 | 0.112 | 0.518 | 0.025 | 0.406 | 0.297 | 0.304 | 0.989 |
| Bucket90 | 3.908  3.914 | 0.399 | 0.799 | 0.814 | 0.308 | 0.681 | 0.112 | 0.181 | 0.118 | 0.955 | 0.221 |
| Bucket91 | 3.968  3.975 | 0.710 | 0.860 | 0.433 | 0.570 | 0.652 | 0.224 | 0.083 | 0.059 | 0.205 | 0.163 |
| Bucket92 | 4.301  4.307 | 0.318 | 0.544 | 0.240 | 0.638 | 0.422 | 0.769 | 0.456 | 0.944 | 0.464 | 0.311 |
| Bucket93 | 4.314  4.321 | 0.544 | 0.666 | 0.638 | 0.814 | 0.518 | 0.389 | 0.481 | 0.767 | 0.966 | 0.318 |
| Bucket94 | 4.424  4.430 | 0.769 | 0.399 | 0.544 | 0.799 | 0.147 | 0.060 | 0.422 | 0.554 | 0.210 | 0.083 |
| Bucket95 | 4.435  4.444 | 0.308 | 0.710 | 0.131 | 0.468 | 0.347 | 0.814 | 0.652 | 0.944 | 0.121 | 0.177 |
| Bucket96 | 4.541  4.550 | 0.308 | 0.203 | 0.468 | 0.799 | 0.232 | 0.153 | 0.622 | 0.046 | 0.059 | 0.168 |
| Bucket97 | 4.550  4.558 | 0.754 | 0.389 | 0.544 | 0.953 | 0.681 | 0.875 | 0.789 | 0.725 | 0.517 | 0.735 |
| Bucket98 | 4.566  4.573 | 0.290 | 0.624 | 0.531 | 0.969 | 0.875 | 0.922 | 0.456 | 0.456 | 0.545 | 0.573 |
| Bucket99 | 4.573  4.580 | 0.378 | 0.969 | 0.695 | 0.652 | 0.337 | 0.724 | 0.346 | 0.855 | 0.704 | 0.683 |
| Bucket100 | 4.589  4.595 | 0.906 | 0.922 | 0.557 | 0.597 | 0.176 | 0.456 | 0.955 | 0.545 | 0.353 | 0.398 |
| Bucket101 | 5.196  5.203 | 0.147 | 0.224 | 0.938 | 0.784 | 0.493 | 0.799 | 0.978 | 0.081 | 0.517 | 0.877 |
| Bucket102 | 5.296  5.316 | 0.337 | 0.170 | 0.875 | 0.299 | 0.953 | 0.583 | 0.311 | 0.088 | 0.757 | 0.632 |
| Bucket103 | 5.330  5.352 | 0.570 | 0.724 | 0.531 | 0.597 | 0.799 | 0.984 | 0.456 | 0.331 | 1.000 | 0.536 |
| Bucket104 | 5.352  5.381 | 0.456 | 0.071 | 0.456 | 0.814 | 0.769 | 0.891 | 0.383 | 0.078 | 0.564 | 0.346 |
| Bucket105 | 5.391  5.456 | 0.085 | 0.610 | 0.531 | 0.754 | 0.518 | 0.769 | 0.800 | 0.243 | 0.554 | 0.602 |
| Bucket106 | 5.478  5.499 | 0.610 | 0.922 | 0.814 | 0.754 | 0.922 | 0.681 | 0.439 | 0.844 | 0.978 | 0.602 |
| Bucket107 | 5.648  5.736 | 0.531 | 0.433 | 0.493 | 0.014 | 0.104 | 0.875 | 0.125 | 0.933 | 0.693 | 0.593 |
| Bucket108 | 5.759  5.770 | 0.347 | **0.009** | 0.131 | 0.399 | 0.071 | 0.210 | 0.237 | 0.248 | 0.757 | 0.663 |
| Bucket109 | 5.770  5.782 | 0.938 | 0.481 | 0.597 | 0.544 | 0.327 | 0.597 | 0.855 | 0.499 | 0.855 | 0.989 |
| Bucket110 | 5.857  5.868 | 0.666 | 0.256 | 0.610 | 0.318 | 0.531 | 0.953 | 0.811 | 0.921 | 0.811 | 0.652 |
| Bucket111 | 5.894  5.901 | 0.984 | 0.922 | 0.638 | 0.318 | 0.147 | 0.531 | 0.414 | 0.536 | 0.683 | 0.456 |
| Bucket112 | 5.901  5.909 | 0.784 | 0.544 | 0.570 | 0.906 | 0.799 | 0.544 | 0.789 | 0.833 | 0.151 | 0.390 |
| Bucket113 | 5.909  5.919 | 0.583 | 0.367 | 0.666 | 0.096 | 0.117 | 0.531 | 0.778 | 0.800 | 1.000 | 0.855 |
| Bucket114 | 5.919  5.927 | 0.433 | 0.224 | 0.170 | 0.281 | 0.597 | 0.096 | 0.822 | 0.989 | 0.422 | 0.899 |
| Bucket115 | 5.927  5.936 | 0.984 | 0.739 | 0.710 | 0.984 | 0.327 | 0.681 | 0.573 | 0.966 | 0.888 | 0.714 |
| Bucket116 | 6.024  6.048 | 0.624 | 0.557 | 0.531 | 0.860 | 0.248 | 0.739 | 0.888 | 0.844 | 0.346 | 0.499 |
| Bucket117 | 6.090  6.114 | 0.481 | 0.518 | 0.189 | 0.597 | 0.081 | 0.666 | 0.353 | 0.800 | 0.439 | 0.318 |
| Bucket118 | 6.130  6.155 | 0.969 | 0.170 | 0.290 | 0.624 | 0.104 | 0.290 | 0.714 | 0.046 | 0.186 | 0.147 |
| Bucket119 | 6.191  6.200 | 0.038 | 0.131 | 0.906 | 0.938 | 0.724 | 0.922 | 0.978 | 0.057 | 0.844 | 0.855 |
| Bucket120 | 6.257  6.277 | 0.248 | 0.240 | 0.984 | 0.557 | 0.240 | 0.681 | 0.297 | 0.009 | 0.573 | 0.545 |
| Bucket121 | 6.280  6.319 | 0.210 | 0.085 | 0.724 | 0.299 | 0.666 | 0.232 | 0.260 | 0.034 | 0.324 | 0.266 |
| Bucket122 | 6.367  6.384 | 0.299 | 0.860 | 0.468 | 0.652 | 0.570 | 0.468 | 0.602 | 0.877 | 0.704 | 0.346 |
| Bucket123 | 6.406  6.416 | 0.638 | 0.695 | 0.638 | 0.891 | 0.232 | 0.122 | 0.536 | 0.490 | 0.989 | 0.473 |
| Bucket124 | 6.552  6.566 | 0.969 | 0.240 | 0.410 | 0.891 | 0.681 | 0.445 | 0.844 | 0.767 | 0.464 | 0.944 |
| Bucket125 | 6.574  6.580 | 0.493 | 0.468 | 0.891 | 0.969 | 0.906 | 0.544 | 0.622 | 1.000 | 0.526 | 0.545 |
| Bucket126 | 6.580  6.593 | 0.969 | 0.906 | 0.984 | 0.518 | 0.092 | 0.281 | 0.811 | 0.430 | 0.398 | 0.499 |
| Bucket127 | 6.595  6.616 | 0.624 | 0.531 | 0.196 | 0.739 | 0.153 | 0.290 | 0.390 | 0.375 | 0.221 | 0.086 |
| Bucket128 | 6.626  6.639 | 0.799 | 0.969 | 0.953 | 0.272 | 0.583 | 0.754 | 0.481 | 1.000 | 0.439 | 0.822 |
| Bucket129 | 6.663  6.675 | 0.938 | 0.891 | 0.505 | 0.108 | 0.337 | 0.136 | 0.430 | 0.855 | 0.318 | 0.147 |
| Bucket130 | 6.773  6.787 | 0.327 | 0.410 | 0.217 | 0.710 | 0.481 | 0.248 | 0.554 | 0.693 | 0.266 | 0.074 |
| Bucket131 | 6.822  6.841 | 0.969 | 0.433 | 0.281 | 0.248 | 0.597 | 0.652 | 0.291 | 0.811 | 0.304 | 0.693 |
| Bucket132 | 6.842  6.848 | 0.014 | 0.922 | 0.065 | 0.695 | 0.624 | 0.367 | **0.025** | 1.000 | 0.714 | 0.056 |
| Bucket133 | 6.925  6.958 | 1.000 | 0.583 | 0.544 | 0.136 | 0.217 | 0.065 | 0.121 | 0.602 | 0.508 | 0.100 |
| Bucket134 | 7.091  7.134 | 0.422 | 0.610 | 0.272 | 0.784 | 0.681 | 0.518 | 0.248 | 0.833 | 0.490 | 0.231 |
| Bucket135 | 7.134  7.144 | 0.078 | **0.024** | 0.875 | 0.410 | 0.357 | 0.953 | 0.800 | 0.260 | 0.602 | 0.642 |
| Bucket136 | 7.524  7.561 | 0.075 | 0.100 | 0.799 | 0.147 | 0.399 | 0.281 | 0.725 | 0.899 | 0.642 | 0.517 |
| Bucket137 | 7.706  7.740 | 0.399 | 0.158 | 0.136 | 0.308 | 0.347 | 0.196 | 0.383 | 0.602 | 0.151 | 0.049 |
| Bucket138 | 7.740  7.752 | 0.327 | 0.984 | 0.147 | 0.153 | 0.518 | 0.544 | **0.021** | 0.683 | 0.989 | 0.195 |
| Bucket139 | 7.879  7.939 | 0.597 | 0.666 | 0.493 | 0.347 | 0.557 | 0.158 | 0.304 | 0.304 | 0.163 | 0.139 |
| Bucket140 | 8.142  8.157 | 0.399 | 0.710 | 0.493 | 0.308 | 0.710 | 0.104 | 0.353 | 0.266 | 0.151 | 0.125 |
| Bucket141 | 8.181  8.202 | 0.433 | 0.652 | 0.829 | 0.784 | 0.481 | 0.422 | 0.966 | 0.693 | 0.612 | 0.554 |
| Bucket142 | 8.202  8.223 | 0.108 | 0.217 | 0.583 | 0.060 | 0.210 | 0.122 | 0.181 | **0.017** | 0.833 | 0.508 |
| Bucket143 | 8.309  8.376 | 0.378 | 0.399 | 0.739 | 0.389 | 0.050 | 0.203 | 0.221 | 0.714 | 0.061 | 0.564 |
| Bucket144 | 8.453  8.468 | 0.557 | 0.445 | 0.754 | 0.531 | 0.557 | 0.248 | 0.481 | 0.464 | 0.215 | 0.564 |
| Bucket145 | 8.616  8.627 | 0.938 | 0.860 | 0.695 | 0.784 | 0.845 | 0.531 | 0.866 | 0.593 | 0.693 | 0.536 |

**Supplementary Table 2:** Pathway analysis between study participants at follow-up visit after six months (visit 2) either receiving a mixture of *Lactiplantibacillus plantarum* HEAL9 and *Lacticaseibacillus paracasei* 8700:2 (intervention) or placebo using genus *Escherichia coli* as a representation of the gut microbiome. Only significant pathways are reported. Raw p-values and p-values adjusted after the Benjamini-Hochberg correction are stated.

| **Pathway Name** | **Match**  **Status*** | **Raw**  **p-value** | **-log(p)** | **Adj.**  **p-value** | **Impact**** |
| --- | --- | --- | --- | --- | --- |
| [Glycine, serine and threonine metabolism](https://new.metaboanalyst.ca/MetaboAnalyst/Secure/pathway/PathResultView.xhtml) | [4/33](https://new.metaboanalyst.ca/MetaboAnalyst/Secure/pathway/PathResultView.xhtml) | 0.013 | 1.89 | 0.270 | 0.34 |
| [Cyanoamino acid metabolism](https://new.metaboanalyst.ca/MetaboAnalyst/Secure/pathway/PathResultView.xhtml) | [2/17](https://new.metaboanalyst.ca/MetaboAnalyst/Secure/pathway/PathResultView.xhtml) | 0.024 | 1.63 | 0.270 | 0.00 |
| [Methane metabolism](https://new.metaboanalyst.ca/MetaboAnalyst/Secure/pathway/PathResultView.xhtml) | [3/26](https://new.metaboanalyst.ca/MetaboAnalyst/Secure/pathway/PathResultView.xhtml) | 0.025 | 1.59 | 0.270 | 0.17 |
| [Pyruvate metabolism](https://new.metaboanalyst.ca/MetaboAnalyst/Secure/pathway/PathResultView.xhtml) | [3/26](https://new.metaboanalyst.ca/MetaboAnalyst/Secure/pathway/PathResultView.xhtml) | 0.027 | 1.57 | 0.270 | 0.11 |
| [Cysteine and methionine metabolism](https://new.metaboanalyst.ca/MetaboAnalyst/Secure/pathway/PathResultView.xhtml) | [3/40](https://new.metaboanalyst.ca/MetaboAnalyst/Secure/pathway/PathResultView.xhtml) | 0.032 | 1.48 | 0.270 | 0.14 |
| [Nicotinate and nicotinamide metabolism](https://new.metaboanalyst.ca/MetaboAnalyst/Secure/pathway/PathResultView.xhtml) | [1/15](https://new.metaboanalyst.ca/MetaboAnalyst/Secure/pathway/PathResultView.xhtml) | 0.049 | 1.31 | 0.284 | 0.00 |

*Fraction of recorded metabolites to the total number of compounds in the pathway.

**Magnitude of altered metabolites´ influence on the specific pathway.

**Supplementary Figures:**


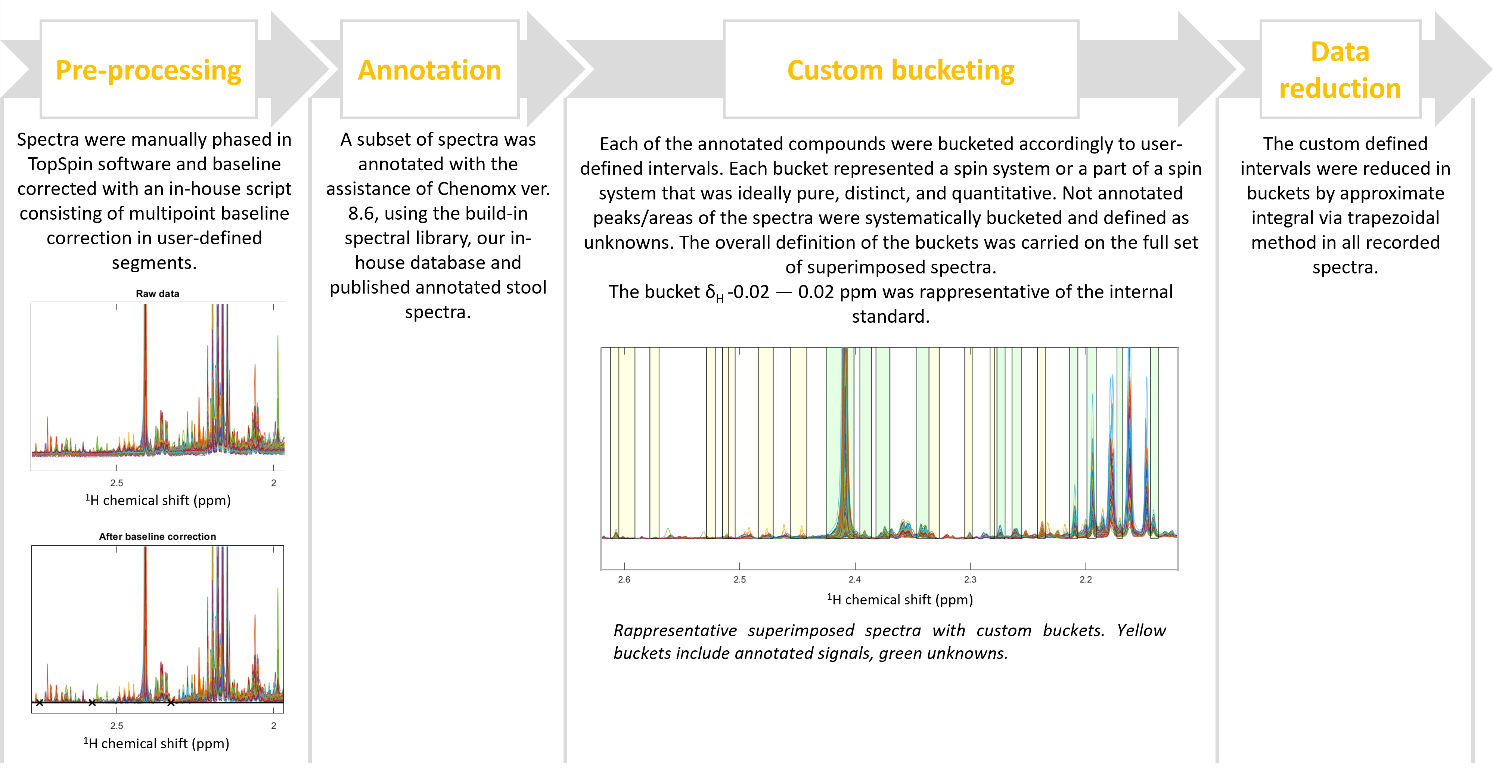


**Supplementary Figure 1:** Scheme of the spectra processing workflow


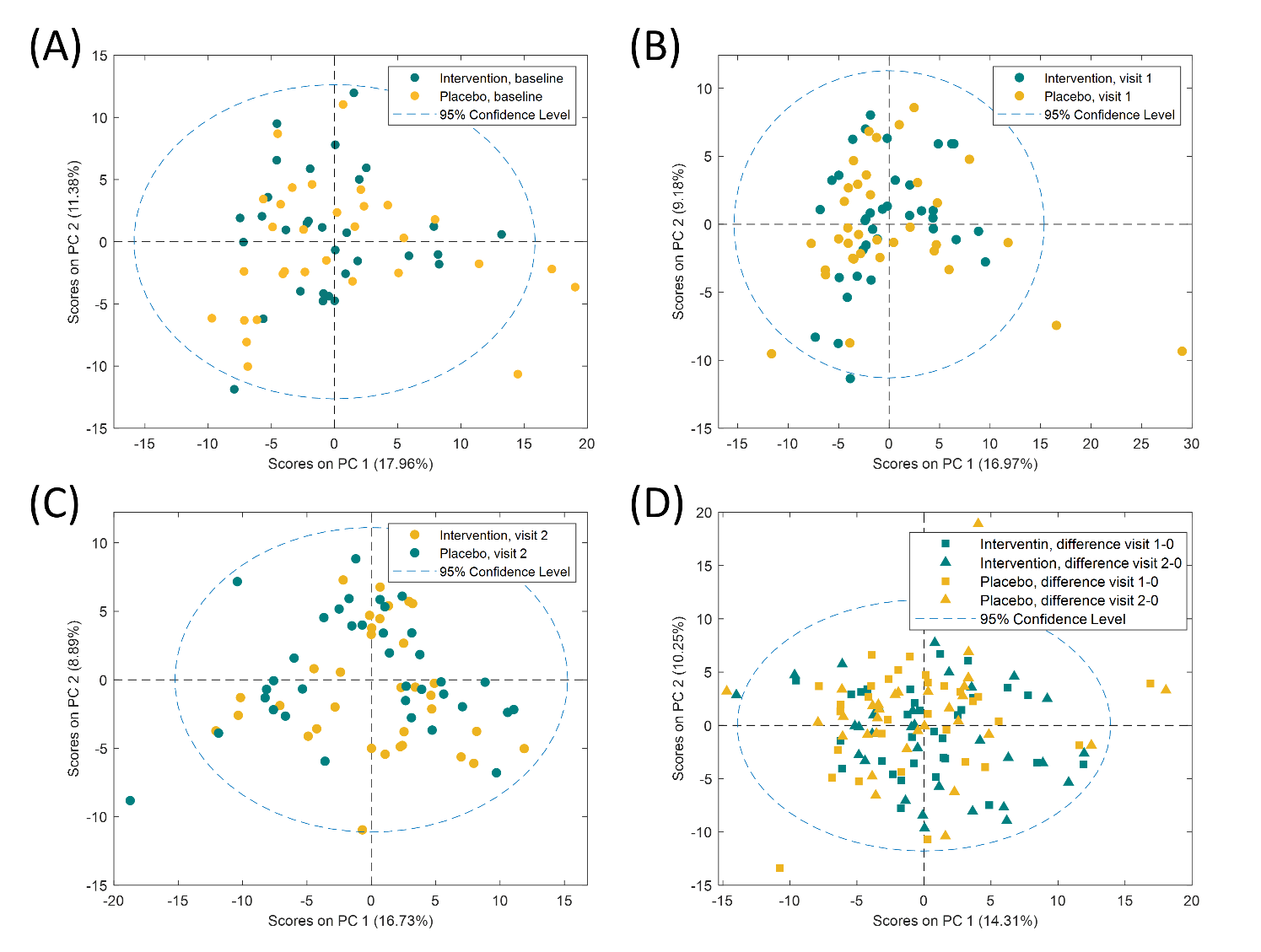


**Supplementary Figure 2:** Principal component analysis for faecal metabolites between study participants either receiving a mixture of *Lactiplantibacillus* *plantarum* HEAL9 and *Lacticaseibacillus* *paracasei* 8700:2 (intervention) or placebo at baseline (A), scheduled follow-up visits after three months (visit 1, B) and six months (visit 2, C) and visit1 and 2 after having subtracted the concentrations registered at baseline on a subject-by-subject basis (D).


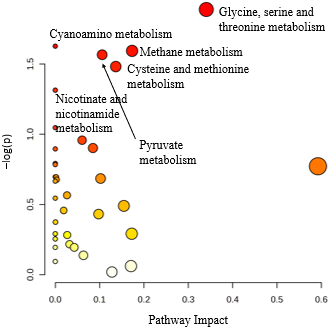


**Supplementary Figure 3:** Pathway analysis between study participants at follow-up visit after six months (visit 2) either receiving a mixture of *Lactiplantibacillus plantarum* HEAL9 and *Lacticaseibacillus paracasei* 8700:2 (intervention) or placebo using genus *Escherichia coli* as a representation of the gut microbiome.
